# Principles of Management of Severe Hyponatremia

**DOI:** 10.1161/JAHA.112.005199

**Published:** 2013-02-22

**Authors:** Antonios H. Tzamaloukas, Deepak Malhotra, Bradley H. Rosen, Dominic S. C. Raj, Glen H. Murata, Joseph I. Shapiro

**Affiliations:** 1 Raymond G. Murphy Veterans Affairs Medical Center, University of New Mexico School of Medicine, Albuquerque, NM (A.H.T., B.H.R., G.H.M.); 2 Department of Medicine, University of Toledo College of Medicine, Toledo, OH (D.M.); 3 George Washington University, Washington, DC (D.C.R.); 4 Marshall University, Huntington, WV (J.I.S.)

**Keywords:** hyponatremia, hypovolemia, osmotic myelinolysis, water diuresis

## Introduction

Hyponatremia represents a serious health hazard.^1^ Hospitalized patients,^2^ nursing home residents,^3^ women,^4–5^ and children^6^ exhibit high frequency and/or severity of hyponatremia. Hyponatremia developing during the course of other morbid conditions increases their severity.^7–10^ Estimates of direct costs for treating hyponatremia in the United States ranged between $1.61 and $3.6 billion.^11^

Clinical manifestations of hyponatremia are universal^12–13^ and range from subtle (disturbances of balance, problems in cognition detected only during specific testing) to life‐threatening manifestations of increased intracranial pressure with life‐threatening hypoxia^14–16^ and noncardiac pulmonary edema.^17^ Although the treating physicians must make an accurate diagnosis based on well‐established and described clinical criteria,^1^ treatment is also guided by the severity of these manifestations. The magnitude and rate of increase in serum sodium concentration ([Na]) during treatment are critical. Overcorrection of chronic hyponatremia may lead to osmotic myelinolysis,^18–21^ whereas undercorrection may fail to prevent life‐threatening manifestations.^1,22^

The mainstays of treatment are restricted free water intake and saline infusion, with or without furosemide. There are 2 indications for saline infusion in hyponatremia. Overt manifestations of hyponatremia are treated with hypertonic saline, whereas symptomatic hypovolemia associated with hyponatremia without overt symptoms is usually treated with isotonic saline.^23–24^ In both situations, the infusion of saline results in rising [Na]. This rise can be slower or faster than desired, with potentially dire clinical consequences.^1,25^

To achieve the desired rise in [Na], several formulas, most often the Adrogue–Madias formula,^23^ are used to calculate volume, rate, and strength of saline infusion. The predictive accuracy of the Adrogue–Madias formula is, in general, good.^26^ However, the rise in [Na] exceeds the value predicted by this formula in some instances, particularly in patients with hypovolemic hyponatremia.^26–27^

This report presents the principles of management of hyponatremia with saline infusion. We analyzed factors that cause deviations in the change of [Na] from the predicted values. We present a clinical protocol for managing hyponatremia with saline infusion based on this analysis.

## Management Principles

**Figure 1.**
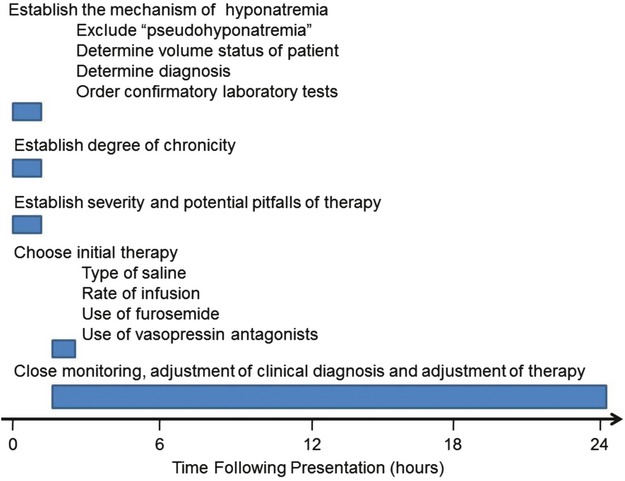
Clinical approach to hyponatremia shown as a flow diagram after initial presentation. Note that the authors recommend making an initial diagnosis and choice of therapy within 2 to 3 hours after presentation with careful monitoring and therapeutic adjustments made thereafter.

Figure 1 shows the application of the principles of management in a flow diagram. All principles are critical for optimizing successful patient outcomes. The principles addressing diagnosis are covered elsewhere.^1,15–16,20^ We have chosen to focus on the principles addressing the quantitative aspects of management in this report.

### Pathogenetic Mechanism, Chronicity

Establishing the pathogenetic mechanism of hyponatremia requires a detailed history that includes medications and drinking habits, physical examination with emphasis on neurological and respiratory signs and on volume status, and serum plus urine laboratory testing. The first step in the differential diagnosis consists of eliminating hypertonic hyponatremia and pseudohyponatremia.^1,15,23,28^

True (hypotonic) hyponatremia results from inability to excrete water loads, usual or excessive. Serum vasopressin is higher than is appropriate for the [Na] in most instances.^29^ Hyponatremia with inappropriately high serum vasopressin levels can be hypovolemic (ie, body water losses relatively lower than sodium losses), euvolemic (ie, body water excess often with some sodium loss), or hypervolemic (ie, water gain in excess of sodium gain).^29^

Hypovolemic hyponatremia presents special challenges. Previous diagnosis of a hypervolemic state, such as congestive heart failure, complicates the diagnosis. The pattern of urinary chemistries (low sodium concentration and high osmolality) is indistinguishable between hypovolemic hyponatremia from extrarenal causes and hypervolemic hyponatremia.^29^ Both conditions lead to vasopressin secretion.^30^ The differential diagnosis is based on careful history and clinical examination. Cautious volume replacement may help when the diagnosis of hypovolemia is doubtful. Thirst from hypovolemia may increase the water load, and alterations in renal circulation may contribute to the decreased renal ability to excrete water.

From a pathophysiological perspective, the loss of brain organic osmolytes occurs with greater chronicity of hyponatremia.^31–32^ Unfortunately, this cannot be determined using existing clinical tools, but a recognition of this fact is essential in understanding potential deleterious aspects of treatment. History, prior measurements of [Na], and the neurological picture at presentation are the only available clinical criteria for determining chronicity. Acute hyponatremia exhibits pronounced brain cell swelling and more severe symptoms but lower risk of osmotic myelinolysis after rapid correction of the [Na], compared with chronic hyponatremia with a similar [Na] value. It is believed that the risk of myelinolysis is greatest where organic osmolyte recovery lags,^31^ and in humans, this area is usually the pons. However, chronic hyponatremia can cause severe neurological manifestations.^33^ When doubt exists, it is safer to consider hyponatremia as chronic.

### Severity

Hyponatremia is considered as severe if [Na] is <115 or 110 mmol/L.^34^ In addition, all cases of hyponatremia treated with hypertonic or isotonic saline infusion, including hypovolemia with hyponatremia and absence of overt neurological manifestations, should be considered as severe because of the risks from saline infusion. Saline infusion for hypovolemic hyponatremia carries arguably the highest risk of inadvertently rapid rise in[Na].

### Target Serum Sodium Concentration

The targeted rise in [Na] depends on the perceived urgency of treatment. In patients with pronounced hyponatremic symptoms, regardless of chronicity, a rapid rise of 4 to 6 mEq/L is recommended.^35^ Further rises may be required if symptoms persist after the initial rise in [Na]. For chronic hyponatremia, previous recommendations set a maximal rate of rise in [Na] at 12 mEq/L in the first 24 hours and a maximal final [Na] of 125 to 130 mEq/L.^34^ Because osmotic myelinolysis was observed in patients achieving the desired rate of rise in [Na],^36^ the current target rise in [Na] is set at 6 to 8 mEq/L in 24 hours, 12 to 14 mEq/L in 48 hours, and 14 to 16 mEq/L in 72 hours.^35^ Prevention of hypernatremia during treatment of hyponatremia is imperative.^37^

### Sodium Concentration and Volume of Infused Saline

Table 1 shows the symbols for volumes and concentrations used in this report. Sodium concentration in commercial saline solutions represents 2 hypertonic (0.855 and 0.513 mol/L), 1 “isotonic” (0.154 mol/L), and 3 “hypotonic” (0.130, 0.077, and 0.034 mol/L) values.^23^ Sodium concentration in the infusate is usually 0.513 mol/L for hyponatremia with pronounced symptoms^35^ and 0.154 mol/L for volume replacement in patients with symptomatic hypovolemia.^29^

**Table 1.** Symbols and Interpretations

Symbol	Interpretation
*V*D_5_W	Volume of 5% dextrose in water
*V*_Inf_	Volume of infused saline
*V*_Lost_	Volume of water lost externally through the skin, the respiratory system, the gastrointestinal system, and the lungs
TBW_I_	Initial (preinfusion) total body water
[Na]_Inf_	Sodium concentration in the infusate
[Na]_Ini_	Initial (preinfusion) serum sodium concentration
[Na]_Fin_	Final (postinfusion) serum sodium concentration
[Na]_Lost_	Average sodium concentration in *V*_Lost_ (the sum of the amounts of sodium lost through the skin, gastrointestinal tract, and kidneys over *V*_Lost_)
[K]_Lost_	Average potassium concentration in *V*_Lost_ (the fraction amount of potassium lost though the skin, gastrointestinal tract, and kidneys over *V*_Lost_)
Na_e_	Total body exchangeable sodium
K_e_	Total body exchangeable potassium
TBW	Total body water
[Na]_pw_	Sodium concentration in plasma water

The sodium concentration in the infusate should not be limited by the strength of the commercial saline solutions. Mixing of saline and dextrose in water can produce any desired sodium concentration by the use of formula 1 (Table 2), which can be of help in hypovolemic hyponatremia with minimal hyponatremic symptomatology, when a large volume of infusate must be reconciled with the need to produce only a modest rise in [Na]. A suitable sodium concentration of the infusate in this instance could be the target [Na] at 24 hours. For example, the target [Na] at 24 hours would be 117 mEq/L in a patient with hypovolemic hyponatremia and initial [Na] of 111 mEq/L. By formula 1, the addition of 0.316 L of dextrose in water to 1 L of 0.154 mol/L saline produces a sodium concentration of 117 mEq/L in the infusate.

**Table 2.** Formulas

Volume of 5% dextrose that needs to be added to 1 L of 0.154 mol/L saline to produce a desired sodium concentration, <154 mEq/L, of the infusate: 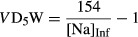 1
Required amount of saline, older formula 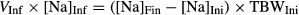 2
Required volume of infusate, older formula 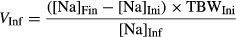 3
The Adrogue–Madias formula^15^ 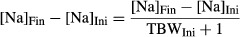 4
Sodium conservation with infusion of any amount of saline into a closed system: 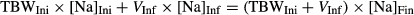 5
Required saline volume (new formula derived from formula 5): 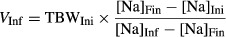 6
Final [Na] (new formula derived from formula 5)*: 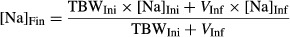 7
The Edelman formula^15^ 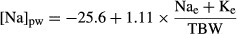 8
Final serum sodium concentration after correction for the osmotic coefficient of infused nonisotonic saline and for external losses of water and electrolytes: 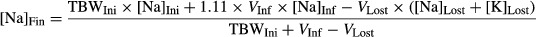 9

*V*D_5_W indicates volume of 5% dextrose in water; [Na]_Inf_, sodium concentration in the infusate; *V*_Inf_, volume of infused saline; [Na]_Fin_, final (postinfusion) serum sodium concentration; [Na]_Ini_, initial (preinfusion) serum sodium concentration; TBW_Ini_, initial (preinfusion) total body water; [Na]_pw_, sodium concentration in plasma water; *V*_Lost_, volume of water lost externally; [Na]_Lost_, average sodium concentration in *V*_Lost_; [K]_Lost_, average potassium concentration in *V*_Lost_.

* If the infused volume is 1 L, the Adrogue–Madias formula is derived by subtracting [Na]_Ini_ from the expression of [Na]_Fin_ in formula 7.

Volume, strength, and rate of saline infused are determined by the symptoms of hyponatremia or hypovolemia and the presenting [Na]. In the past, the required amount and volume of hypertonic saline were calculated by formulas 2 and 3 (Table 2), which do not take into account the effect of infused water on the change in [Na]. The Adrogue–Madias formula^23^ (formula 4 in Table 2), which calculates the predicted change in [Na] after infusion of 1 L of saline, accounts for the major factors that determine the changes in [Na] after the addition of saline to a closed system (initial [Na] and body water plus sodium concentration and volume of the infused saline). Not accounting for the water infused has caused errors in calculations of the changes in [Na] resulting from hypertonic infusions in experimental settings.^38–39^ The magnitude of the error increased as the infused volume increased.

Although formula 4 represents a conceptual improvement in the prediction of changes in [Na] after saline infusion, it cannot compute directly the amount of saline required for a desired rise in [Na] or the predicted rise in [Na] after infusion of a volume of saline that is not a multiple of 1 L. To address these issues, we developed formulas 5 to 7 (Table 2) accounting for the same factors as the Adrogue–Madias formula.

### Representative Patient

**Figure 2.**
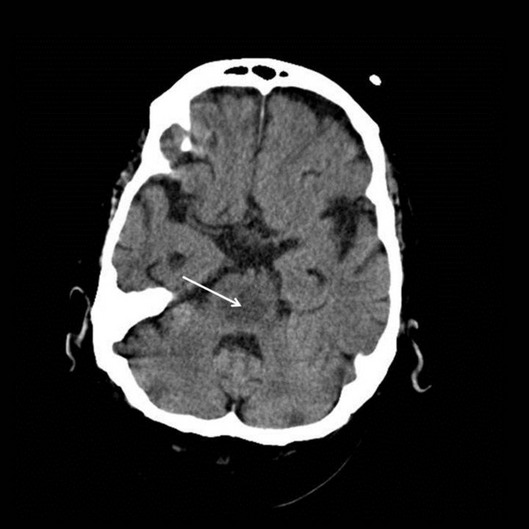
Magenetic resonance imaging brain slice from index patient showing myelinolysis in pons (*white arrow*).

To illustrate quantitative differences between measured and formula‐predicted [Na] values after saline infusion and the contributions to these differences by various factors affecting the accuracy of the predictive formulas, Table 3 presents details of a patient with hypovolemic hyponatremia who developed after saline infusion overcorrection of [Na] and osmotic myelinolysis. A slice of this patient's brain magnetic resonance image is shown to illustrate this myelinolysis (Figure 2).

**Table 3.** Representative Patient With Hypovolemic Hyponatremia

	Baseline	First Infusion	Second Infusion
TBW_Ini,_* L	26		
TBW_Ini,_* L	36		
V_Inf_, 0.154 mol/L saline, L		1.75	0.75
Infusion duration, h		6	12
[Na]_Ini_, mEq/L	111.0		
Actual [Na]_Fin_, mEq/L		120.0	129.0
Predicted [Na]_Fin_,** mEq/L		121.4	125.8
Predicted [Na]_Fin_,** mEq/L		113.7	114.8
Predicted [Na]_Fin_,** mEq/L		118.5	121.7
Predicted [Na]_Fin_,** mEq/L		113.0	113.8
Predicted [Na]_Fin_,*** mEq/L		116.0	117.5

The patient was a man with left above the knee amputation; at presentation, age 55 years, height 157.5 cm, weight 60 kg. TBW_Ini_ indicates initial (preinfusion) total body water; *V*_Inf_, volume of infused saline; [Na]_Ini_, initial (preinfusion) serum sodium concentration; [Na]_Fin_, final (postinfusion) serum sodium concentration; GI, gastrointestinal.

* TBW_Ini_ calculated from the anthropometric anthropometric Watson formula^40^ corrected for the effects of above‐the‐knee amputation^41–42^ and for the magnitude of volume depletion estimated from the change in serum albumin concentration before and after treatment.^43^

* TBW_Ini_ calculated as 60% of presenting weight.

* From formula 2 solved for [Na]_Fin_.

* From formula 7.

* From formula 9, assuming that (1) respiratory loss of oxygen was doubled from normal because of the persistent hyperventilation (arterial p_CO2_ was in the range of 20 to 22 mm Hg in 3 measurements during the first 3 days of hospitalization), rising the estimated loss of water during the first infusion of saline through the lungs, skin and GI tract from 0.188 to 0.288 L) and (2) losses through the skin, gastrointestinal tract, and kidneys were negligible. Urine sodium concentration was 10 mEq/L and urine osmolality was 74 mOsm/kg at the end of the first infusion.

#### Estimates from various formulas

**Figure 3.**
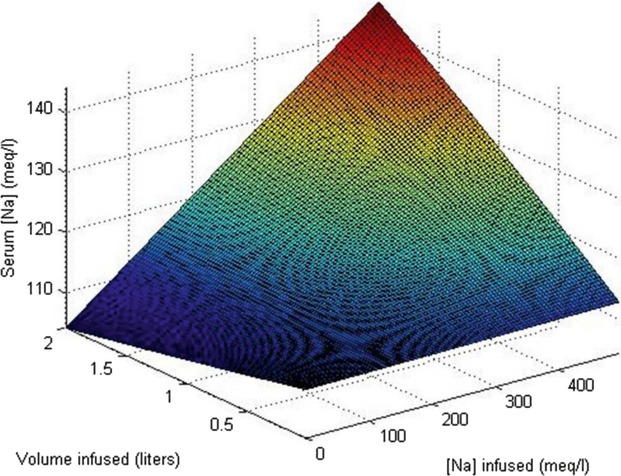
Serum sodium concentration changes ([Na]) after infusion of 1.75 L of saline with varying sodium concentration in a patient with initial body water of 26 L and initial [Na] of 111 mEq/L. The changes in [Na] were computed by formula 7 of this report.

For these estimates, initial [Na] was considered as equal to 111 mEq/L and initial body water as 26 L. Figure 3 shows [Na] changes after infusion of varying volumes of saline with varying sodium concentration predicted by formula 7. If potassium salts are also infused, the sum of sodium plus potassium concentration in the infusate should be substituted for sodium concentration in formulas 6 and 7.

Table 4 shows volumes of 0.154 mol/L saline required to raise [Na] to 117 mEq/L calculated by formulas 2, 4, and 6 in Table 3. Formula 4 requires 5 steps to calculate a desired volume of the infusate between 4 and 5 L. In first step, this calculated volume is 4.21 L by formula 6 but only 1.01 L by formula 2. Comparison of these predictions to the findings of Table 3 shows that formula 2 overestimated, while formulas 4 and 6 underestimated, the rise in [Na] after the first saline infusion.

**Table 4.** Calculation, by Various Formulas From Table 2, of the Volume of 0.154 mol/L Saline Required for an Increase in Serum Sodium Concentration From 111 to 117 mEq/L in a Patient With 26 L of Initial Body Water

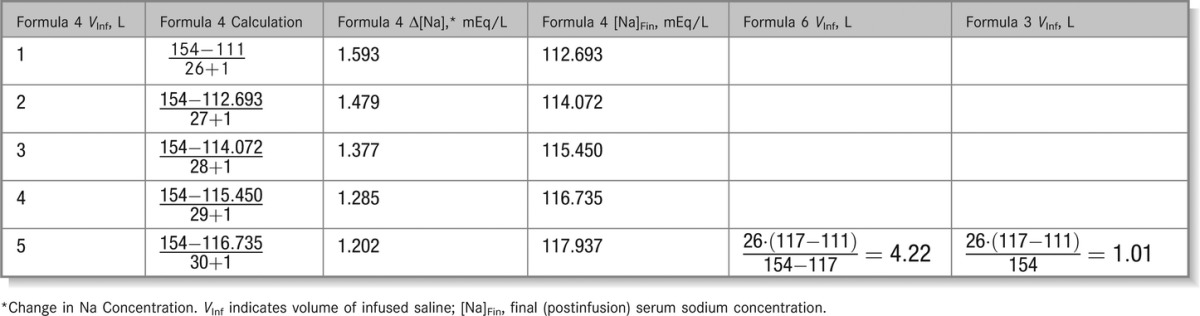

### Pitfalls of the Formulas for Saline Infusion

The potential pitfalls of formulas 2 to 7 include inaccuracies of estimates entered in the formulas, inaccuracies of the formulas, and problems caused by assuming a closed system.

#### Inaccuracies of estimates entered in the formulas

**Figure 4.**
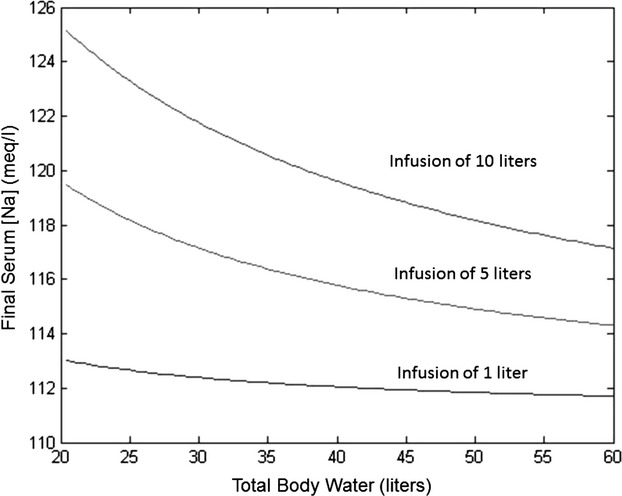
Effect of varying body water estimates on the change in serum sodium concentration of a patient with initial serum sodium concentration ([Na]) of 111 mEq/L infused with various volumes of 0.154 mol/L saline. Calculations from formula 7 (Table 3).

Among these estimates, sodium concentration in serum and infusate and volume of infusate can be accurately measured, but clinical estimates of body water with adjustments for volume abnormalities^1^ are essentially inaccurate. Figure 4 shows that the predicted effect of widely varying estimates of body water on the changes in [Na] after infusion of various volumes of 0.154 mol/L saline is relatively small. After the first infusion of saline in the illustrative patient, predicted by formula 7, [Na] values differed by only 0.7 mEq/L, whereas initial body water estimates differed by 10 L; both substantially lower than the observed [Na] value (Table 3). Although variation in the estimates of body water has a small effect on the discrepancies between observed and predicted [Na], it is appropriate to use in the calculations realistic values for body water, especially in hyponatremia with pronounced hypovolemia when lower values of body water produce higher estimates of the postinfusion [Na].

#### Inaccuracies of predictive formulas

Formulas 2 to 7 do not take into account several factors potentially affecting [Na], including changes in body content of solutes other than sodium or potassium, in exchangeable potassium and sodium from body pools not readily available for rapid changes in osmolality, in plasma water content, and in the osmotic coefficients of sodium and potassium salts, plus effects of the Gibbs–Donnan equilibrium.^40^ Kurtz and Nguyen^40^ suggested that the cumulative effects of all the influences on sodium concentration in plasma water are shown in the formula of Edelman^41^ (formula 8 in Table 2). The effects on [Na] of these other factors can be substantial in states other than true hyponatremia, such as in hypertonic hyponatremia.^42^ Areas requiring exploration are slow changes in cell volume after rapid osmotic changes^43^ and changes in body sodium pools not readily available for osmotic regulation^43–45^ and in intracellular solutes other than sodium or potassium induced by potassium deficits.^46^

#### Problems caused by assuming a closed system

Formulas 2 to 7 do not account for changes in body sodium, potassium, or water other than saline infusion. Under experimental conditions mimicking closed systems, formulas similar to formula 7 predicted accurately the changes in [Na] after the induction of hypernatremia^39,47^ or hyponatremia.^46,48^ However, patients with dysnatremia do not represent closed systems. They exhibit external losses of solute and water during treatment. The magnitude of these losses, which are usually hypotonic, varies depending on the pathogenetic mechanisms of the dysnatremia, the effects of treatment on these mechanisms, and other conditions present.

Losses occur through the respiratory system, the skin, the gastrointestinal track and the kidneys. Normally, average loss of water though the first 3 routes is ≈1100 mL (≈400 mL through the lungs, ≈500 mL through the skin, and ≈200 mL through the gastrointestinal system), whereas water generation from oxidation amounts to 350 mL per 24 hours. Net water loss amounts to 750 mL per 24 hours or 188 mL per 6 hours.^49^ Loss of solute through the 3 routes is proportionally lower than water loss. Sodium concentration in sweat is 30 to 65 mEq/L.^49^ In the stool, average sodium concentration is 40 mEq/L, and potassium, 90 mEq/L.^50^ Water and solute losses increase in sweating, vomiting, or diarrhea and hyperpnea. Urinary losses vary during treatment of hyponatremia. After correction of uncomplicated hypovolemia, urine flow increases as the volume stimulus for vasopressin secretion disappears and water diuresis ensues. Overcorrection of hyponatremia may follow.^25–27^

Formula 9 in Table 2 calculates the final [Na] in patients infused with saline after correcting the osmotic coefficient of the infused saline^40–41^ and taking into account losses of water, sodium, and potassium through all 4 external routes. Formula 9 and similar formulas accounting for external losses^51–52^ can be used to validate the principals involved in their development by post facto observation, as was done recently in experimental acute hyponatremia.^53^ Another use of these formulas is in illustrating the quantitative effects of each of the factors affecting the change in [Na] during saline infusion (see later examples). However, the magnitude of external losses cannot be predicted at the onset of treatment. Consequently, calculation of the amount of infused saline is done with closed systems formulas.

**Figure 5.**
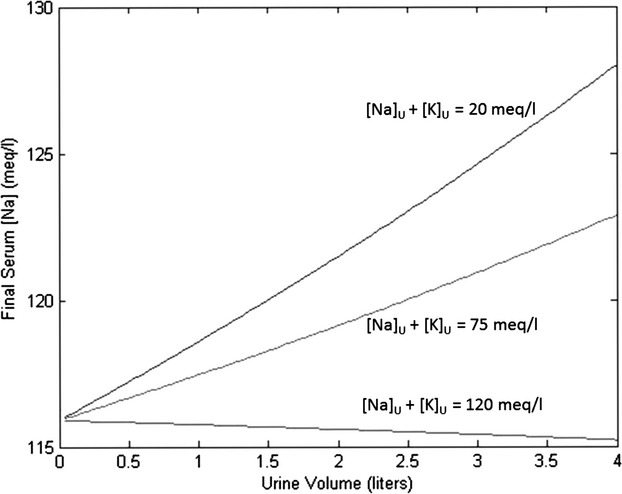
Effect of urine composition ([Na]_U_+[K]_U_) and flow rate on serum sodium concentration [Na] after infusion of 1.75 L of 0.154 mol/L saline in a patient with initial body water of 26 L and [Na] of 111 mEq/L calculated from formula 9 (Table 3) if all the other influences depicted in this formula except urinary losses result in an [Na] of 116.0 mEq/L (Table 5). At [Na]_U_+[K]_U_=116 mEq/L, urinary losses have no effect on [Na]. [Na] decreases if [Na]_U_+[K]_U_ >116 mEq/L and increases if [Na]_U_+[K]_U_ <116 mEq/L.

In the illustrative patient with initial body water of 26 L, formula 9 computes that infusion of 1.75 L of 0.154 mol/L saline would cause a rise in [Na] from 111 mEq/L to 116.0 mEq/L if through the lungs, skin, and gastrointestinal tract loss of water was 0.288 mL and losses of sodium and potassium were negligible during the first saline infusion (Table 3). Figure 5 shows predicted changes in [Na] from diuresis in this patient. The direction of the change in [Na] is determined by the sum of urine sodium and potassium concentrations. Regardless of the urine volume, [Na] will be equal to the predicted value of 116 mEq/L if the sum of urine plus potassium concentration is equal to 116 mEq/L. For the same urine volume, the lower the sum of urinary sodium plus potassium, the greater the rise in [Na] will be. The volume of urine containing 10 mEq/L each of sodium and potassium needed to raise [Na] to 120 mEq/L after infusion of 1.75 L of 0.154 mol/L saline is 1.1 L.

Modest hypotonic urine production can cause large underestimates of the increase in [Na] during treatment of hyponatremia with saline. External losses, primarily though the urine during treatment of hypovolemic hyponatremia, represent the major pitfall of formulas 2 to 7.

## Management Protocol

Table 5 summarizes the management of hyponatremia with saline infusion. Closed system formulas (formulas 2 to 7) provide estimates of the required saline volume and allow comparison between desired and observed changes in [Na] and, therefore, provide the frame for identifying the source of their deviations and the guide for appropriate treatment changes. The first aim of treatment is to avoid undercorrection of hyponatremia.^5^ Prescription of the volume of saline infused by formula 4 or 6 is suitable for this purpose. Monitoring is critical when saline is infused, particularly in hypovolemic hyponatremia in which water diuresis, overestimation of initial body water, and initial focusing on volume rather than tonicity issues complicate the treatment. Monitoring, with reduced frequency of [Na] measurement (usually once daily), is essential during treatment of hyponatremia without saline infusion.

**Table 5.** Steps of the Management of Severe Hyponatremia

1. Evaluation of pathogenesis and chronicity History, physical examination, temporal evolution of [Na] before presentation
2. Establishment of indications for saline infusion—determination of severity Clinical manifestations of hyponatremia or [Na] <115 mEq/L Clinical manifestations of hypovolemia
3. Collection of baseline information required for saline infusion Body weight Serum electrolytes, glucose, urea nitrogen, creatinine, osmolality, albumin Other serum values (uric acid, cortisol, thyroid hormones, etc), as needed Urine sodium and potassium concentrations and osmolality
4. Calculation of the volume, strength, and rate of saline infusion using formula 6 (Table 2) (a) For symptomatic hyponatremia: hypertonic saline (b) For symptomatic hypovolemia with low presenting [Na]: isotonic saline or calculation of salinity using formula 1 (Table 2) (c) For a combination of (a) and (b): initially hypertonic saline, followed by saline with [Na] concentration computed from formula 1
5. Continuous monitoring throughout the infusion—intensive care unit preferred Clinical: neurological status, respiration, volume status every 2 to 3 hours; body weight daily, and more frequently if needed Urine flow rate: hourly Serum: sodium and potassium every 2 to 3 hours; other values (osmolality, urea nitrogen, glucose) as needed; creatinine daily; albumin at the end of the infusion Urine: sodium, potassium every 6 hours or more frequently if urinary flow rate increases during the infusion; osmolality if needed
6. Changes in the management Comparison of actual and predicted (from formula 7, Table 2) [Na]_Fin_ after each measurement of [Na] Evaluation of causes of discrepancy (formula 9) Addition of furosemide to the infusion, taking care that the rate of saline infusion exceeds the rate of urine flow in patients with hypovolemic hyponatremia Infusion of hypotonic saline or vasopressin plus water

[Na]_Fin_ indicates final (postinfusion) serum sodium concentration.

Many tests, especially urine chemistries and osmolality, cannot be obtained rapidly from all hospital laboratories. For this and other reasons, administration, along with saline, of loop diuretics (eg, furosemide) to make urine free water excretion more predictable may be helpful in managing hypovolemic hyponatremia. Although furosemide will initially increase urinary sodium and potassium excretion, it is reasonable to assume that the sum of urine sodium plus potassium concentration is equal to ≈75 mEq/L when a furosemide effect is present,^1,54^ at least until direct laboratory measurements are available. Because patients with hypovolemic hyponatremia have reduced total body sodium and probably water, care must be taken to replace more than the predicted urinary electrolyte and water losses with infused saline.

Vasopressin V2 receptor antagonists may ultimately be extremely useful for treating complicated chronic hyponatremias.^55^ However, it is unclear how to best use these new agents at present. It is fair to say that the vasopressin V2 receptor antagonists appear to be very effective in the settings of heart failure, cirrhosis, and syndrome of inappropriate antidiuretic hormone secretion and safe when administered as monotherapy.^56–57^ Unfortunately, these agents are currently extremely expensive. Moreover, we would stress that these agents should be avoided during saline infusion to prevent the hazards of excessive water diuresis.

## Conclusion

Accurate diagnosis of the cause, pathogenesis and chronicity, and monitoring during treatment are the critical parts of the management of severe hyponatremias. We stress that calculation errors are possible even with the best formulas, and frequent monitoring of the patient during therapy is absolutely essential to ensure optimal chances for recovery.
